# Finger Nails

**Published:** 1855-12

**Authors:** 


### FINGER NAILS

GROW out about three times a year; they should be trimmed with scissors once a week, not so close as to leave no room for the dirt to gather, for then they do not protect the ends of the fingers, as was designed by nature; besides, if trimmed too close at the corners, there is danger of their growing into the flesh, causing inconvenience, and sometimes great pain.

The collections under the ends of the nails should not be removed by anything harder than a brush or a soft piece of wood; nor should the nails be scraped with a penknife or other metallic substance, as it destroys the delicacy of their structure, and will at length give them an unnatural thickness. We are not favorably impressed as to the cleanliness of a person who keeps his nails trimmed to the quick, as it is often done to prevent dirt gathering there; whereas, if a margin were allowed, it would be an index to the cleanliness of the hands, from which the collections under the finger nails are made. Leave a margin, then, and the moment you observe that these collections need removal, you may know that the hands need washing, when they and the nails are both cleaned together.

Most persons are familiar with those troublesome bits of skin which loosen at the root of the finger nails; it is caused by the skin adhering to the nail, which, growing outward, drags the skin along with it, stretching it until one end gives way. To prevent this, the skin should be loosened from the nail once a week, not with a knife or scissors, but with something blunt, such as the end of an ivory paper cutter; this is best done after soaking the fingers in warm water, then pushing the skin back gently and slowly; the white specks on the nails are made by scraping the nail with a knife at the point where it emerges from the skin.

Biting off the finger nails is an uncleanly practice, for thus the unsightly collections at the ends are kept eaten clean! ! Children may be broken of such a filthy habit by causing them to dip the ends of their fingers several times a day in wormwood bitters, without letting them know the object; if this is not sufficient, cause them to wear caps on each finger until the practice is discontinued.

